# Laparotomic myomectomy for a huge cervical myoma in a young nulligravida woman: A case report and review of the literature

**DOI:** 10.18502/ijrm.v18i2.6421

**Published:** 2020-02-27

**Authors:** Hatem Abu Hashim, Moustafa Al Khiary, Mohamed EL Rakhawy

**Affiliations:** ^1^Department of Obstetrics and Gynecology, Faculty of Medicine, Mansoura University, Mansoura, Egypt.; ^2^Department of Diagnostic Radiology, Faculty of Medicine, Mansoura University, Mansoura, Egypt.

**Keywords:** Cervix, Fibroid, Leiomyoma, Myomectomy.

## Abstract

**Background:**

A huge cervical myoma (rare) in a young woman is a nightmare of every gynecologist owing to the associated technical challenges in performing a myomectomy. Moreover, the 2014 US Food and Drug Administration prohibited power morcellation during laparoscopic myomectomy due to the inadvertent spread of occult malignancy and an increased risk of iatrogenic parasitic leiomyoma negatively affected the overall rate of a minimally invasive surgery.

**Case:**

This report described our experience with a case of a huge anterior cervical myoma (473 gr) in a young nulligravida woman who successfully underwent laparotomic myomectomy. After an initial diagnosis by Magnetic resonance imaging (MRI), we performed preoperative ureteric catheterization. The myoma was enucleated following the footsteps of Victor Bonney, the pioneer of myomectomy, combined with simple additional steps. We did not use preoperative gonadotropin-releasing hormone analog, intraoperative vasopressin injection, or uterine artery ligation. A 6-month follow-up MRI revealed an intact cervical canal in midline position with no evidence of residual fibroid.

**Conclusion:**

Based on our experience, the review of the relevant literature, and the US Food and Drug Administration's prohibition of power morcellation during laparoscopic myomectomy, a laparotomic myomectomy for a huge cervical myoma still plays a vital role in fertility preservation. We propose the mnemonic "MUSIC" as a helpful guide for a consistent strategy: M (preoperative MRI), U (prophylactic ureteric catheterization), S (shell out the myoma following Bonney's principles i.e. start-up and stay intracapsular), I (immediate suction to clarify dead space) and C (close the cavity by spiraling stitch).

## 1. Introduction

Cervical myomas are rare and accounts for 0.6% of all uterine fibroids (1). Magnetic resonance imaging (MRI) is the eminent modality for its detection. Large cervical fibroids could be identified on T2-weighted sagittal imaging and categorized as either extra cervical (i.e., subserosal that may be anterior, posterior, or lateral) or intracervical (i.e., intramural) (2). Unlike corporeal fibroids, cervical myomas commonly present with lower abdominal pain and symptoms of pelvic pressure, such as frequent urination, constipation, and sometimes, dyspareunia (3). Unlike vaginal myomectomy for prolapsed cervical fibroids, abdominal cervical myomectomy was practically absent from the literature until the last decade (4). Narrow operative field, possible injuries to surrounding pelvic structures, such as ureters, urinary bladder, and rectum, significant hemorrhage, and a difficult repair of the big cavity render the procedure technically challenging (3). Recently, the US Food and Drug Administration prohibited the retrieval of myoma by power morcellation during laparoscopy (5).

The primary objective of this report is to describe our experience with a case of a huge anterior cervical myoma in a young nulligravida woman who successfully underwent laparotomic myomectomy to preserve her fertility. In addition, we carried out a review of the relevant literature.

## 2. Case Report 

### Patient information and clinical findings

A 27-year-old nulligravida woman was presented to our hospital with a four-month history of lower abdominal pain, frequent urination, and dyspareunia. She had regular menstrual cycle without any menstrual-related complaints. In addition, there were no constitutional symptoms or bowel complaints. Her medical and surgical history was non-eventful and there was no family history of cancer. The general examination did not detect any abnormality. Her body mass index was 21.7. Liver and spleen were not palpable abdominally, but there was a firm non-tender pelvic–abdominal mass about the size of 16 weeks pregnancy with restricted mobility. There was no evidence of ascites. A vaginal examination revealed that a mass was felt anteriorly and through all fornices. Posterior lip of the portion – vaginalis of the cervix was felt as a rim and taken up posteriorly. The mass with the size of about 16 weeks pregnancy with restricted mobility was felt by the bimanual examination. The uterus could not be felt separately. The cervix was not visualized on speculum examination since a bulging mass was observed in the upper portion of vagina.

### Diagnostic assessment 

**Figure 1:**
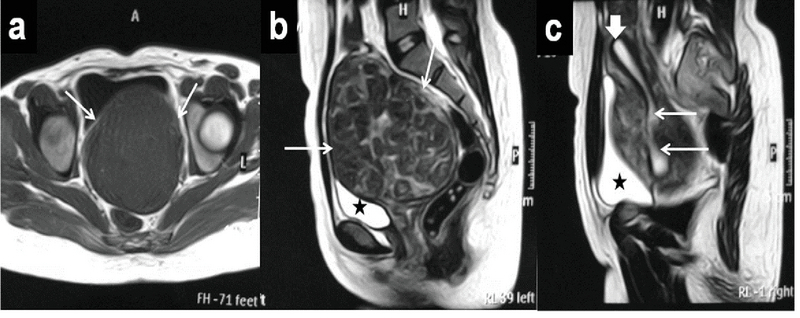
Preoperative MRI of a huge anterior cervical myoma occupying the pelvic cavity in a 27-year-old nulligravida woman, a. Axial T1-weighted MR image shows a low SI of the mass (white arrows), b. Sagittal T2-weighted MR image shows a mixed low and intermediate SI of the mass (white arrows), c. Right parasagittal T2-weighted MR image shows a uterine body on the top of the mass (thick white arrow) with an elongated cervical canal in the posterior aspect of the cervical myoma (thin white arrows), MRI: Magnetic resonance imaging; SI: Signal intensity. ★; Urinary bladder

Her lab profile values were within the normal limits of hemoglobin: 13.3 g/dL. Ultrasonography revealed a huge pelvic mass that measured 11 × 12 cm (probably of uterine origin). MRI revealed a huge anterior cervical myoma measuring 10.5 × 9.2 × 12 cm (anteropsterior, transverse, and height, respectively) with the uterine body visualized above and the fundus reaching 5 cm cranially to the sacral promontory (Figure 1). Neither hydronephrosis nor hydroureter was detected by the IVP; however, the left ureter was mildly and laterally displaced.

### Operative steps

The patient was counseled for surgery with a high possibility of a hysterectomy. Due to financial constraints, she did not receive preoperative gonadotropin-releasing hormone analog (GnRHa). After an informed consent, prophylactic ureteric catheterization was performed. Exploratory laparotomy revealed a huge anterior cervical myoma that measured about 10.5 × 9 × 12 cm filling the pelvis with normal corpus uteri on its top and deviated backward to the right. Both fallopian tubes and ovaries were grossly normal. Bilateral uterine artery ligation (BUAL) at its origin from the internal iliac artery (IIA) was not possible. Myometrial injection of diluted vasopressin was not performed due to non-availability of the drug in our country.

**Figure 2:**
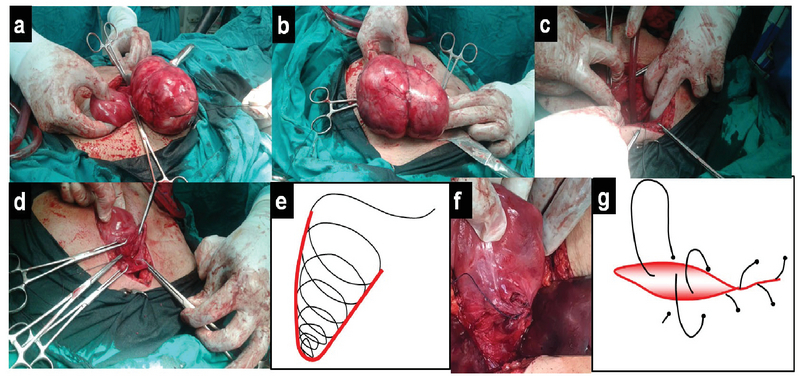
Intraoperative photography of laparotomic myomectomy for a huge anterior cervical myoma in a 27-year-old nulligravida woman. a. Myoma is just attached at the base of its capsule. Traction stitch (silk suture number 1) is observed. b. Myoma is completely enucleated. c. The wound edges are being pulled up by forceps and suction to clarify the tumor bed. d. The cavity is obliterated by continuous concentric layers of "spiraling stitch." e. Schematic presentation of the "spiraling stitch." f. A "baseball stitch" is inserted to close the superficial wound edges after its trimming. g. Schematic presentation of the "baseball stitch."

The peritoneal flap over the tumor was incised at its upper limit and, then, reflected downward. The footsteps of Victor Bonney, the pioneer of myomectomy, were meticulously followed to shell out the myoma (6). First, the capsule of the tumor was incised at its upper portion for about 5 cm. Then, the index finger of the left hand was inserted through the incision to ascertain the plane of cleavage between the tumor and its capsule. Subsequently, the incision was enlarged across the capsule and the tumor was gradually enucleated down to its base by using the fingers of the right hand through the proper plane of cleavage. Due to the non-availability of the myoma screw, enucleation was assisted by inserting a traction silk stitch (number 1) and pulling on it with the left hand (Figure 2a). The base of the capsule was coagulated before the complete enucleation of myoma (Figure 2b). Subsequently, the edges of the capsule were pulled up by forceps with a simultaneous suction to clarify the tumor bed in depth (Figure 2c). The cavity was swiftly obliterated by applying the continuous concentric layers of "spiraling stitch" (7) with 0 polyglactin 910 inserted in a bottom-up manner (Figure 2 d, e). Ureteric catheters were felt before the suture placement at the base of the defect to avoid the ureteric damage during suturing. The first layer placed at the base of the defect was tied. Continuous traction and maintaining constant tension on the suture after each layer was ensured during the whole procedure. Finally, we performed the trimming of redundant wound edges and closure by a "baseball stitch" with 2-0 polyglactin 910 (Figure 2 f, g). A drain was placed within the cul-de-sac. There was an estimated blood loss of approximately 150 mL. The operative time was 80 min. The weight of the excised myoma was 473 gr. The postoperative course was uneventful. After two days, the drain, ureteric and Foley's catheters were removed and the patient was discharged. Cervical leiomyoma was confirmed histopathologically.

### Follow-up and outcomes 

**Figure 3:**
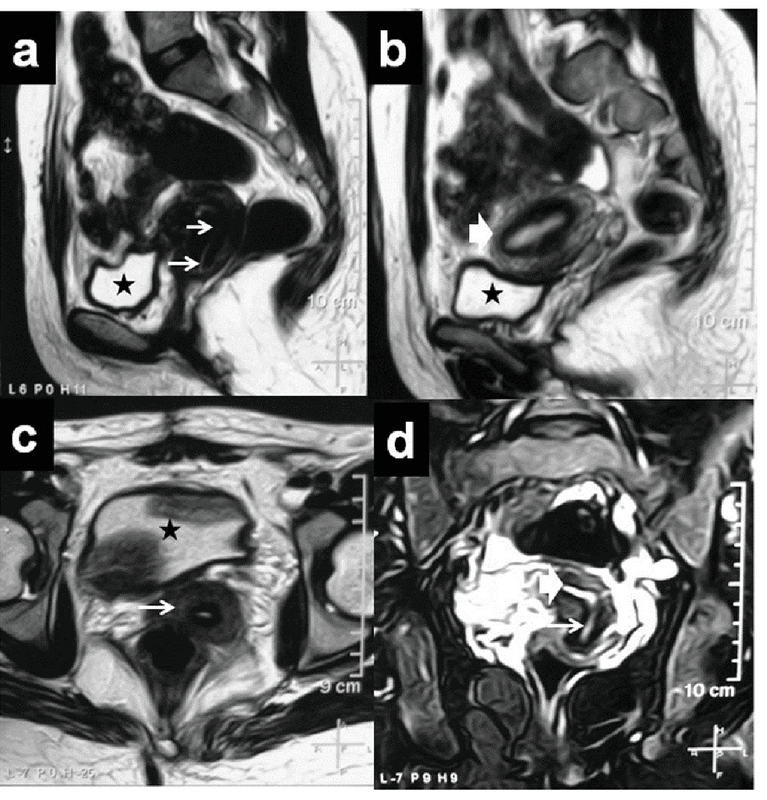
A six-month follow-up MRI. a. Sagittal T2-weighted MR image shows an intact cervical canal in the midline position (white arrows). b. Right parasagittal T2-weighted MR image shows a uterine body (thick white arrow). c. Axial T2-weighted MR image shows the cervix (white arrow) with no evidence of residual fibroid. d. Coronal T2-weighted MR image shows an intact cervical canal in the midline position (thin white arrow) and continuous with the endometrium above (thick white arrow). MRI: Magnetic resonance imaging. ★; Urinary bladder

**Figure 4:**
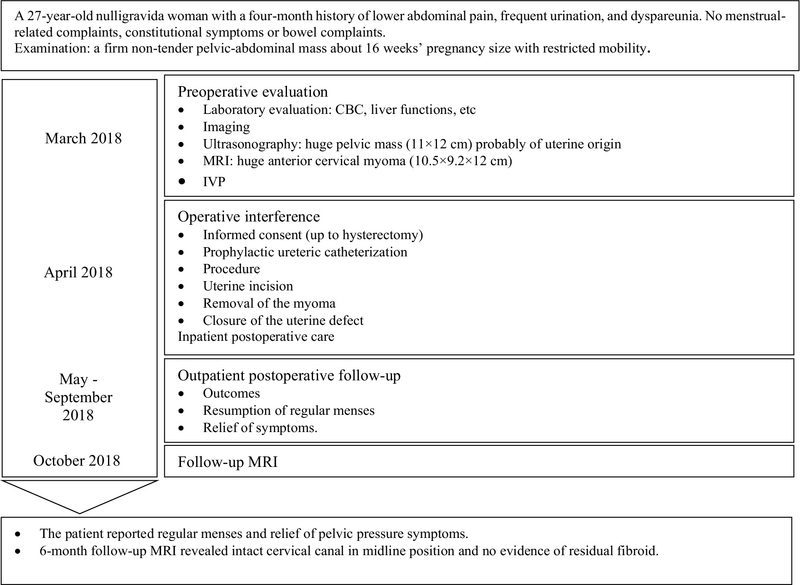
Timeline of preoperative, operative and postoperative care.

The patient was regularly followed up monthly for about six months. Outcomes included the resumption of regular menses and symptom relief. The patient reported regular menses and a relief of pelvic pressure symptoms. A six-month follow-up MRI revealed an intact cervical canal in the midline position with no evidence of residual fibroids (Figure 3). Figure 4 presents the relevant data from this episode of care organized as a timeline.

### Ethical consideration

This case report was approved by the local research ethics committee of our institution (MFM-IRB: Mansoura Faculty of Medicine Institutional Research Board, Code No: R/572/07/2019) and prior written informed consent was obtained from the patient.

## 3. Discussion

Abdominal myomectomy should be the standard fertility preservation procedure of choice for cervical fibroids in women who desire to preserve their fertility. We reviewed the literature with (`cervical myoma' OR `cervical fibroid) AND myomectomy in PubMed. In view of the aforementioned rarity of cervical myomas, it is not surprising to have a limited number of case reports and series of laparoscopic cervical myomectomy (LCM) in which small- and moderate-sized cervical myomas were extirpated by power morcellation. Of note, it appeared in the literature in the last decade, and the detailed information is summarized in Table I.

In our case, prophylactic ureteric catheterization assisted in avoiding the inadvertent ureteric damage during suture placement at the base of the myoma bed. Preoperative GnRHa administration has the merits of fibroid size reduction and improving the hemoglobin level in women with anemia (3). Our patient did not receive GnRHa due to financial constraints, but her basal hemoglobin level was satisfactory (13.3 g/dL). In the largest case series of LCM (28 patients), only 2 patients received preoperative GnRHa (4). The mean weight of all excised myomas was 287 gr, and, it ranged between 250 and 499 gr in five cases (4). The latter is comparable to the excised myoma in our case (473 gr).

Measures utilized to minimize the intraoperative blood loss include BUAL at its origin from the IIA and intraoperative injection of diluted vasopressin into the myoma (4, 8). BUAL at its origin was carried out in two LCM case series (8, 9). The mean maximum diameter of the excised myomas was 8.5 cm and its median weight was 220 gr in one series (9) and ranged between 99.3 and 306.2 gr in the other one (8). In our case, we were not able to perform BUAL at its origin, because the huge fibroid (473 gr) was completely filling the pelvic cavity, thereby restricting access to the pelvic sidewall dissection. This difficulty was also recognized in LCM for the large myomas of greater than 250 gr, and BUAL was only achieved after the downsizing of myoma by partial enucleation and morcellation (4). Unlike other research works (4, 8), the intraoperative injection of diluted vasopressin into the myoma was not carried out in our case due to non-availably of this medication in our country.

The estimated blood loss in our case was 150 mL. Chang and colleagues (4) reported a mean blood loss of 99 mL in his series with the aid of BUAL and diluted vasopressin injection. However, they admitted a loss of up to 500 mL in the case of a huge myoma (1.200 gr). The key principle that prevented massive hemorrhage in our case was the strict application of Bonney's principles to shell out the myoma (i.e., the transverse incision made along its prominent proximal portion and ascertaining the plane of cleavage between the tumor and its capsule) (6). Immediate suction to clarify the dead space and swiftness in its suture closure also reduced the blood loss. Undoubtedly, laparotomy allowed enough working space for traction and counter-traction, thus achieving a rapid enucleation of such huge myoma. Moreover, the spiraling stitch (7) was not only hemostatic but also closed the defect efficiently eliminating any dead space.

The strengths of our approach to this case are that the procedure was carried out successfully without any complications by strictly following the above mentioned footsteps of Victor Bonney, the pioneer of myomectomy, combined with simple additional steps. On the contrary, this approach has several limitations. First, preoperative GnRHa administration was not utilized to reduce the fibroid size. However, financial constraints precluded its usage. Second, the intraoperative injection of diluted vasopressin into the myoma to reduce the blood loss was not performed. However, this is due to the non-availability of this medication in our country. Third, BUAL at its origin from the IIA to reduce the blood loss was not performed. However, the huge fibroid (473 gr) was completely filling the pelvic cavity precluding access to the pelvic sidewall dissection. Finally, it could be argued that LCM may be performed in this case. However, despite all the merits of LCM as a minimally invasive approach, morcellation is still the utilized option for the retrieval of myomas, especially the huge ones. In 2014, the US Food and Drug Administration warned against the use of power morcellation for uterine leiomyomas owing to the concerns about the inadvertent spread of occult malignancy (5). Together with an increased risk of iatrogenic parasitic leiomyoma and disseminated peritoneal leiomyomatosis, these concerns negatively affected the overall rate of minimally invasive surgery (10). Therefore, a laparoscopic myomectomy for our case (myoma of 473 gr) is not advisable because it will not be achieved except after its initial downsizing by partial enucleation and morcellation followed by shelling out of the remaining part and its morcellation.

**Table 1:** Summary of review of the available literature of abdominal cervical myomectomy (only case series and reports)


**Authors (yr) (ref)**	**Country**	**Study type**	**Type of cervical myoma**	**Operation done**	**Details**
**Chang ** ***et al*** **. 2010. (Fertil Steril. 94: 2710-2715)**	Taiwan	Case series (n = 28)	1 (IC) 27 (EC)	Laparoscopic cervical myomectomy BUAL + vasopressin (GnRHa only in 2 patients)	-16 were nulliparous -Mean age of patients 38 (24–52) years -Mean maximum diameter of the myoma by US not reported -Mean operative time: 121 (45–280) min -Mean blood loss: 99 (50–500) mL -Mean specimen weight: 287 (30-1,200) gr
**Matsuoka ** ***et al*** **. 2010 (J Minim Invasive Gynecol. 17: 301-305)**	Japan	Case series (n = 16)	5 (IC) 11 (EC)	Laparoscopic cervical myomectomy GnRHa + BUAL + vasopressin	-7 were nulliparous -Mean age of patients 37.3 (35.2–39.6) years -Mean maximum diameter of the myoma by MRI 6.9 (6.0–7.8) cm -Mean operative time: 105.8 (82.8–128.8) min -Mean blood loss: 105 (42.6–167.4) mL -Mean specimen weight” 208.3 (99.3–306.2) gr
**Sinha ** ***et al*** **. 2009 (J Minim Invasive Gynecol. 16: 604-608)**	India	Case series (n = 12)	Not reported	Laparoscopic cervical myomectomy BUAL (GnRHa and vasopressin not used)	-None were nulliparous -Mean age of patients: 36 (28–43) years -Mean maximum diameter of the myoma by US 8.5 cm -Median operative time: 90 (60–120) min -Median blood loss: 50 (30-100) mL -Median specimen weight: 220 (180–440) gr
**Higuchi ** ***et al*** **. 2012 (Asian J Endosc Surg. 5: 126-130)**	Japan	Case series (n = 8)	5 (Intramural) 1 (Subserosal) 2 (Submucosal)	Laparoscopic cervical myomectomy GnRHa + vasopressin (BUAL not done)	All were nulliparous Data in 7 patients: -Mean age of patients: 35.5 (30–44) years -Mean maximum diameter of the myoma by MRI: 7.6 (5.0–12.8) cm -Mean operative time: 176 (125–255) min -Mean blood loss: 71.4 (30–200) mL -Mean specimen weight: 132 g (16–310) g -One patient, with a large myoma (900 g) was converted to laparotomy due to blood loss (1,800 mL)
**Takeuchi ** ***et al*** **. 2006 (J Minim Invasive Gynecol. 13: 334-336)**	Japan	Case series (n = 5)	IC	Laparoscopic cervical myomectomy (GnRHa + BUAL + + vasopressin)	-4 were nulliparous -Mean age of patients: 36.2 years -Mean maximum diameter of the myoma by MRI 5.8 cm -Mean operative time: 70 min -Mean blood loss: 18 mL -Mean specimen weight: 79.6 gr
**Takeda ** ***et al*** **. 2009 (Fertil Steril. 91: 935. e5-e9)**	Japan	Case report	EC	Laparoscopic- assisted myomectomy (GnRHa + prophylactic temporary endovascular balloon occlusion of the bilateral internal iliac arteries + vasopressin)	-A 33-year-old nulligravida woman -Maximum diameter of the myoma by MRI not reported -Operative time: 130 min -Blood loss <50 mL -Specimen weight: 1,036 g
**Garzon-Lopez ** ***et al*** **. 2015 (J Minim Invasive Gynecol. 22: S218)**	Mexico	Case report (Conference Abstract)	EC.	Laparoscopic cervical myomectomy (vasopressin)	-A 31 year-old woman, para 1 -Maximum diameter of the myoma by MRI: 15 cm -Operative time: 260 min -Blood loss not reported -Specimen weight: 800 gr
**Peng ** ***et al*** **. 2016 (Taiwan J Obstet Gynecol. 55: 293-295)**	Taiwan	Case report	Not reported	Laparotomic cervical myomectomy	-42-year-old gravida2, para 2 -US & Computed topography (CT) 10×7.4 cm pelvic mass -Operative time, blood loss, and specimen weight not reported -Pathology: cervical leiomyoma with myxoid degeneration
**Peker ** ***et al*** **. 2017 (J Minim Invasive Gynecol. 24: 345-346)**	Turkey	Video demonstration of laparoscopic cervical myomectomy	Not reported	Laparoscopic cervical myomectomy (GnRHa, vasopressin, BUAL not done)	-40-year-old nulliparous woman -Maximum diameter of the myoma by US: 14 × 10 cm -Operative time: 140 min -Blood loss: 300 mL -Specimen weight: 670 g
**Tian and Hu 2012 (Aust N Z J Obstet Gynaecol. 52: 258-261)**	China	Case series (n = 17), 5 were nulliparous	Not reported	Cesarean myomectomy In 9 cases	-Mean age of patients: 32.8 (28–41) years -Maximum diameter of the myoma (5 cases 5–10 cm, 2 cases 11–19 cm, 1 case < 5 cm, 1 case ≥ 20 cm) -Mean blood loss: 697 mL (range 350–2,000 mL) -Mean operative time and specimen weight not reported
BUAL: Bilateral uterine artery ligation at its origin from the internal iliac artery; cm: Centimeter; EC: Extracervical (i.e., subserosal); g: Gram; GnRHa: Gonadotropin-releasing hormone analog; IC: Intracervical (i.e., intramural); min: Minute; mL: Milliliter; MRI: Magnetic resonance imaging; n: Number of cases; US: Ultrasonography					

## 4. Conclusion

A laparotomic myomectomy for a huge cervical myoma should be offered as the standard choice if the patient desires to preserve her fertility. It is technically feasible by following the footsteps of Victor Bonney combined with simple additional steps. We propose the mnemonic "MUSIC" as a helpful guide for a consistent surgical strategy: M (preoperative MRI for diagnosis), U (prophylactic ureteric catheterization), S (shell out the myoma following Bonney's principles, i.e., start-up and stay intracapsular), I (immediate suction to clarify dead space), and C (close the cavity by spiraling stitch). The gynecologic surgeon should make a diligent effort in following these technical principles that are the mainstay to avoid intraoperative urological injuries and massive hemorrhage.

##  Conflict of Interest 

The authors have no conflicts of interest relevant to this article.
